# Emergence of a PRRSV Strain Recombined From Two Modified-Live Virus Vaccines and Its Elimination From a Breeding Herd

**DOI:** 10.1155/tbed/5770608

**Published:** 2025-07-20

**Authors:** Giovani Trevisan, Joel Sparks, Michael Zeller, Hao Tong, Ganwu Li, Jianqiang Zhang, Phillip C. Gauger, Christopher Rademacher, Rodger Main, Ana Paula Poeta Silva, Daniel C. L. Linhares

**Affiliations:** ^1^Veterinary Diagnostic and Production Animal Medicine, Iowa State University, Ames, Iowa, USA; ^2^AMVC, Frankfort, Indiana, USA

**Keywords:** animal health, genetic evolution, management practices, production loss, PRRSV, recombination

## Abstract

Porcine reproductive and respiratory syndrome (PRRS) is one of the most challenging diseases for swine production. The PRRS virus (PRRSV) is an RNA virus that replicates via an RNA-dependent RNA polymerase (RDRP) mechanism, which is prone to high mutation rates. Recombinations are characterized by the exchange of genetic material across two or more viruses. Modified live virus (MLV) vaccines produce an immune response to PRRSV after replicating in pigs, similar to natural exposure. Here, we report the emergence of an MLV-like recombinant strain, its associated production impact, and its disappearance trajectory from a breeding herd. The emergent virus was identified and successfully eliminated from a 9248-sow breed-to-wean herd. Accidental usage of two distinct MLVs in the herd led to the recombination and emergence of a new strain. The clinical presentation was mild compared to current wild-type strains, with the associated production loss amounting to 549 weaned piglets per 1000 sows. Production levels returned to normal within 7 weeks. Transitory, no significative production loss in the wean-to-market phase was identified. Immunization of the herd and tightening of biosecurity and biocontainment practices were able to eliminate the virus from the herd, without evidence of broad regional spread.

## 1. Introduction

The report of a Mystery Swine Disease in North America at the end of the 1980s, coupled with similar complaints in Europe, led to the discovery and characterization of a swine disease named porcine reproductive and respiratory syndrome (PRRS) caused by the PRRS virus (PRRSV) [1]. A serological study from stored samples in Canada showed the presence of PRRSV antibodies in 1978 [2]. PRRSV is an RNA virus from the family *Arteriviridae* classified into two distinct species: PRRSV-1 (*Betaarterivirus europensis*), first reported in Europe, and PRRSV-2 (*Betaarterivirus americense*), first reported in North America (International Committee on Taxonomy of Viruses [ICTV], https://ictv.global/report/chapter/arteriviridae/arteriviridae/betaarterivirus). The two species share about 40% nucleotide differences across the whole genome, with both species present in the United States [3–10]. PRRSV continues to cause disease in pigs of all ages, culminating with an annualized economic production loss of $1.2 billion for U.S. commercial swine production [11, 12], with significant losses also reported in Canada and Europe [13–15].

As an RNA virus, PRRSV replicates via an RNA-dependent RNA polymerase (RDRP) mechanism, which is prone to a high mutation rate [16], allowing PRRSV to adapt to environmental changes associated with the development of the immune response by host or pharmacological challenges [16]. Random substitution, deletions, insertions, and recombinations allow an RNA virus to genetically evolve over time and evade the immune system. A recombination event is characterized by the exchange of genetic material between two or more viruses, creating a newly derived virus. To persist in the population, the emerging virus needs to be shed in the environment and infect new susceptible hosts [17–19]. In recent years, the increased availability of next-generation sequencing (NGS) diagnostic testing techniques, reduction of diagnostic testing costs, and improved epidemiological ability to use and interpret the results have allowed the detection of PRRSV recombination events [19–29]. PRRSV recombination events can happen between wild-type, modified live virus (MLV) vaccines or between both. Natural selection limits the ability of all recombinant viruses to survive, establish within a population, and become predominant [16].

PRRS MLV vaccines are genetically characterized with sequences publically available, making the bioinformatics process straightforward to identify recombination events involving MLV-derived viruses. In contrast, not all wild-type PRRSV strains are well characterized by the whole genome due to NGS cost, nor is there a comprehensive and representative database with all historical sequences available, making it difficult to characterize all wild-type recombinant viruses. During vaccine manufacturing, MLV vaccines go through an attenuation process, which is considered safe for use in healthy animals. MLV label recommendation is restricted to use in healthy animals susceptible to PRRSV with the intended outcome of inducing and maintaining immunity for PRRSV, aiding in preventing the respiratory and reproductive distress caused by wild-type PRRSV. However, the MLV strains need to replicate in the host to develop herd immunity. If, for unlabeled reasons, two MLV vaccines replicate in a host at the same time, it opens the opportunity for recombination events, similar to what can happen with wild-type viruses. However, the clinical implications of PRRSV MLV recombinant virus in the field are rarely detected and reported. This work reports the emergence of an MLV-like recombinant strain, its associated production impact, and its disappearance trajectory from a breeding herd.

## 2. Materials and Methods

### 2.1. Herd Demographics and Clinical Investigation

**Figure 1:**
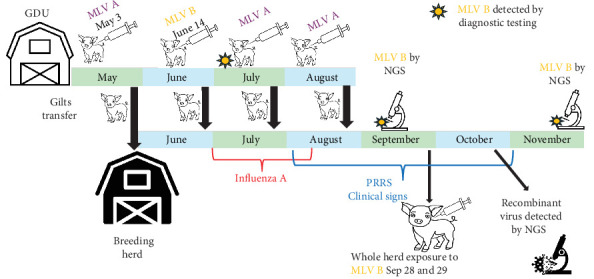
Animal movement, modified live vaccine exposure (MLV), next generation sequencing (NGS) testing sequence of events in the gilt development unit (GDU), and the breeding herd.

An offsite gilt development unit (GDU), housing gilts between 7–30 weeks of age and the sole supplier of replacement gilts to a 9248-head breeding herd, located in the state of Indiana, United States, had gilts routinely exposed at the GDU to PRRSV acclimation with Prevacent PRRS (Elanco Animal Health, Greenfield, IN), a commercially available PRRSV-2 MLV vaccine. Routine exposure occurred 1 day after gilt arrival in GDU, with a Prevacent PRRS administration occurring on May 3, 2023. On June 14, 2023, a different MLV vaccine, Fostera PRRS (Zoetis, Parsippany, NJ), a commercially available PRRSV-2 MLV vaccine, was erroneously ordered by the farm staff through the veterinary clinic and administered to the youngest, 7-week-old, group of gilts in the GDU (Figure 1). Based on the PRRSV ORF5 classifications, Prevacent PRRS (GenBank KU131568) is classified as Lineage [30] 1D, Variant [31] 1D.2, and restriction fragment length polymorphism (RFLP) [32] 1-8-4. Fostera PRRS (GenBank AF494042) is classified as Lineage 8C, Variant 8C.1, and RFLP 1-3-2. On a whole-PRRSV genome basis [21], Prevacent PRRS does not classify according to a lineage, and Fostera PRRS classifies as Lineage 1. In July 2023, monthly PRRSV diagnostic testing revealed the circulation of Fostera PRRS in the GDU, prompting an investigation to identify the issue (Figure 1). At this time, the oldest group of gilts in the GDU, having 25–27 weeks of age, had already been moved to the breeding herd (Figure 1). Gilt acclimation at the GDU continued with Prevacent PRRS (Figure 1).

At the beginning of August 2023, sow mortality increased, and piglet health indicated a mild–moderate PRRSV infection that was additionally complicated by an ongoing influenza A virus outbreak initially diagnosed in July 2023 (Figure 1). For the breeding herd, serum samples were collected monthly from 30 due-to-wean pigs for surveillance testing by PRRSV RT-PCR in pools of 5:1. In mid-September 2023, lung samples from sick pigs were collected PRRSV RT-PCR and NGS tested, and a whole PRRSV genome of 15,363 base pairs (bps) having 99.7% nucleotide identity to Fostera PRRS was recovered (Figure 1). As a countermeasure, the whole breeding herd was exposed to Fostera PRRS from September 28^th^ to 29^th^, 2023 (Figure 1). Even though sow mortality had improved within a month, the PRRSV viremia in weaned piglets and the quality of piglets remained problematic. Clinical evaluation by the attending veterinarian reported poor performance and fuzzy pigs, decreased weight gain, and early farrows resulting in low birth weight, and low-viability pigs. Postweaned pigs had a typical clinical PRRS appearance. On October 30, 2023, the sera pool with the lowest Ct was subjected to ORF5 sequencing via PRRSV Fostera MLV ORF5 CLAMP Sanger method, PRRSV whole genome sequencing using NGS, and virus isolation using MARC-145 cells [33, 34]. On November 27^th^, 2023, the pool with a PCR Ct of 23.5 was sequenced by NGS, recovering a whole PRRSV genome of 15,298 bps having 99.54% nucleotide identity with Fostera PRRS (Figure 1).

### 2.2. PRRSV Plaque Purification

PRRSV was successfully isolated in MARC-145 cells from a sera pool collected on October 30, 2023, and the obtained PRRSV isolate was subjected to plaque purification. To conduct a PRRSV plaque purification, the virus was 10-fold serially diluted (10^−1^–10^−6^) in the RPMI 1640 cell culture medium supplemented with 10% fetal bovine serum (FBS), 2 mM L-glutamine, 0.05 mg/mL gentamicin, 100 unit/mL penicillin, 100 mg/mL streptomycin, and 0.25 mg/mL amphotericin. After removing the culture medium from MARC-145 cell monolayers grown in 6-well plates, 200 µL of each virus dilution was added to individual wells. The plates were incubated at 37°C with 5% CO_2_ for 1 h, with gentle rocking every 15 min to keep the monolayer moist. After incubation, the virus inoculum was removed, and 2 mL of warm overlay medium (RPMI 1640 medium supplemented with 2% low-melting-point agarose and 2% FBS) was added to each well. Once the overlay medium solidified, the plates were incubated at 37°C with 5% CO_2_, and plaque formation was monitored daily. When distinct plaques became visible at some dilutions, four plaques were selected using pipette tips. Each selected plaque was transferred to confluent MARC-145 cell monolayers in a 6-well plate and incubated at 37°C with 5% CO_2_. Upon observation of ~80% of cytopathic effects, the cultures underwent one freeze–thaw cycle. The resulting cell lysates were centrifuged at 2,000 rpm for 10 min, and the supernatants were collected and stored at −80°C. Eventually, two plaque-purified virus samples were tested for whole-genome sequencing via NGS.

### 2.3. PRRSV Recombination Analysis and Phylogenetic Tree Construction

The PRRSV whole genome recovered from the October 30, 2023 sera pool (Genbank PV607082) and the duplicated plaque-purified virus (GenBank PV607080 and PV607081) were further used to investigate a recombination event between Prevacent PRRS (GenBank KU131568) and Fostera PRRS (GenBank AF494042). The recovered genome and the MLV sequences were aligned using MAFFT v7.450 default settings [35]. RDP4 was used to detect recombination events that were considered if supported by at least six of the seven default parameters available in the software [36]. The recombination event was confirmed and visualized through Simplot [37].

A whole genome phylogenetic tree containing the recombinant strain, parental and additional MLV strains, and representative wild-type PRRSV-2 strains was constructed. Publicly available sequences were downloaded from GenBank. The PRRSGard MLV and a Lineage 1C.2 sequence were provided by Iowa State University Veterinary Diagnostic Laboratory. Sequences were aligned using MAFFT and provided as input to build a neighbor-joining consensus tree using Geneious Tree Builder (Geneious Prime 2025.1.3, https://www.geneious.com), setting the Tamura–Nei genetic distance with a bootstrap resampling method run with 100 replications. The phylogenetic tree was rooted in the PRRSV-2 reference strain VR-2332.

### 2.4. Quantification of Production Loses in the Breeding Herd and Growing Sites

Production impact associated with the emergence of the new virus was measured in the breeding herd as total piglet losses per 1000 sows (TL), which is calculated by taking the total number of piglets not weaned after the outbreak, divided by inventory, and then multiplied by 1000 in order to standardize it per 1000 sows. The week of August 6^th^, 2023, after the erroneous administration of Fostera PRRS in the GDU and introduction of gilts to the breeding herd, was used as week zero, and the 21 weeks preceding this were used to establish farm-specific baselines for weekly total pigs weaned. The last 2 weeks of July and the first week of August were removed from baseline due to an influenza A virus outbreak in the breeding herd. An exponential weighted moving average (EWMA) with parameters set to 3*σ* to adjust the width of upper and lower control limits and *λ* of 0.4 for assigning weight to the most recent variable value included in the data [38] was used for the total pig weaned monitoring during the upcoming 40 weeks. Deviation from baseline was considered when the EWMA dropped below the lower confidence limits (LCLs). Returning to baseline was considered when the EWMA returned to the LCL and stayed within the confidence limit boundaries for at least 2 weeks. TL was calculated as the cumulative sum of the number of pigs not weaned below mean baseline levels per 1000 sows until the herd returned to baseline production.

Similarly, growing phase mortality, comprising the period from weaning to the market of weaned piglets placed in wean-to-finish barns, was assessed and compared before and after the detection of MLV recombinant virus. Following the procedure used in the breeding herds, the week of August 6^th^, 2023, was used as week zero, and wean-to-finish placed groups between May 25, 2023 to end of July were used to establish flow-specific wean-to-market baselines for total cumulative closeout mortality, i.e., total deads during the wean-to-market phase divided by the number of places pigs. Pigs placed in the last 2 weeks of July up to the week of August 6^th^ were removed from the baseline and control period due to the ongoing influenza A virus circulating in the breeding herd. The EWMA was set with the same 3*σ* and *λ* of 0.4 parameters, as for the total pig weaned in the breeding herd, for monitoring the mortality during the upcoming 40 weeks after the week of August 6^th^. Growing mortality differences before and after the introduction of a second MLV and detection of a recombination event, i.e., before and after week zero, were assessed using a generalized linear mixed model using PROC GLIMMIX in SAS 9.4 (SAS Institute Inc., Cary, North Carolina). The wean-to-finish cumulative mortality counts were the outcome of interest, i.e., the dependent variable, assuming a Binomial distribution, and the closeouts group as the explanatory variable in the model. The groups placed before August 6 were set as referents.

### 2.5. Assessing the Regional Spread of the Recombinant Virus

The production system considered wean-to-finish placed lots as positive for PRRSV and did not perform additional downstream PRRSV ORF5 sequencing. Regional spread of the recombinant PRRSV was assessed by searching the recombinant ORF5 sequence through the Swine Disease Reporting System Blast Database (SDRS, https://fieldepi.org/sdrs/blast-tool/), considering similar sequences as having ≥99% nucleotide identity level. SDRS was chosen to assess the regional spread since it aggregates PRRSV ORF5 sequences in real-time from participant VDLs representing >98% of PRRSV ORF5 sequencing done in the U.S. National Animal Health Laboratory Network [39].

## 3. Results

**Figure 2:**
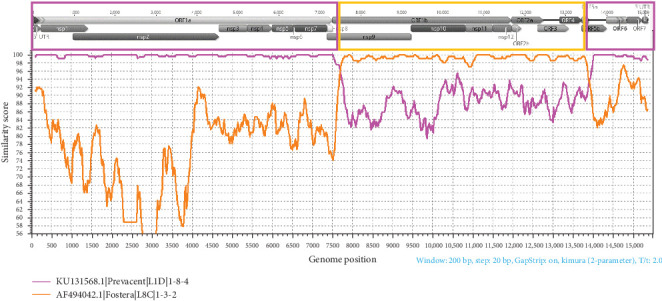
Similarity plot between a PRRSV recombinant virus derived from two modified live virus vaccine strains. PRRSV genome regions are presented at the top of the plot. Purple and orange boxes outline the recombination breakpoints and genome regions that were derived from each of the parental PRRSV vaccine strains.

The sera collected in October 2023 from due-to-wean piglets were positive for PRRSV by RT-PCR with Ct values ranging from 16.1 to 23.5 (average 19.7). PRRS Fostera CLAMP was positive, i.e., no Fostera PRRS vaccine virus was detected, and Sanger recovered a PRRSV ORF5 sequence with 98% nucleotide identity with Prevacent PRRS and 88.9% with Fostera PRRS. The pooled sample with a Ct of 16.1 was subjected and positive to PRRSV isolation in MARC-145 cells. NGS performed on the isolate recovered a PRRSV genome of 15,010 bp. An additional contig of 3540 bp having 99% nucleotide identity, and comprising positions 291–1340 of an alignment with Fostera PRRS was also recovered. The recovered whole genome appeared to have two recombination breakpoints: position 7616 within the nonstructural protein 9 of the ORF1b and position 13,912 located within the ORF5. Nucleotides 1–7615 and 13,913–15,477 were derived from the major parental Prevacent PRRS strain, whereas nucleotides 7616–13,912 were derived from the minor parental strain Fostera PRRS (Figure 2). The ORF5 sequence determined via Sanger methods and the ORF5 sequence extracted from the whole genome recovered by NGS had 100% nucleotide identity.

**Figure 3:**
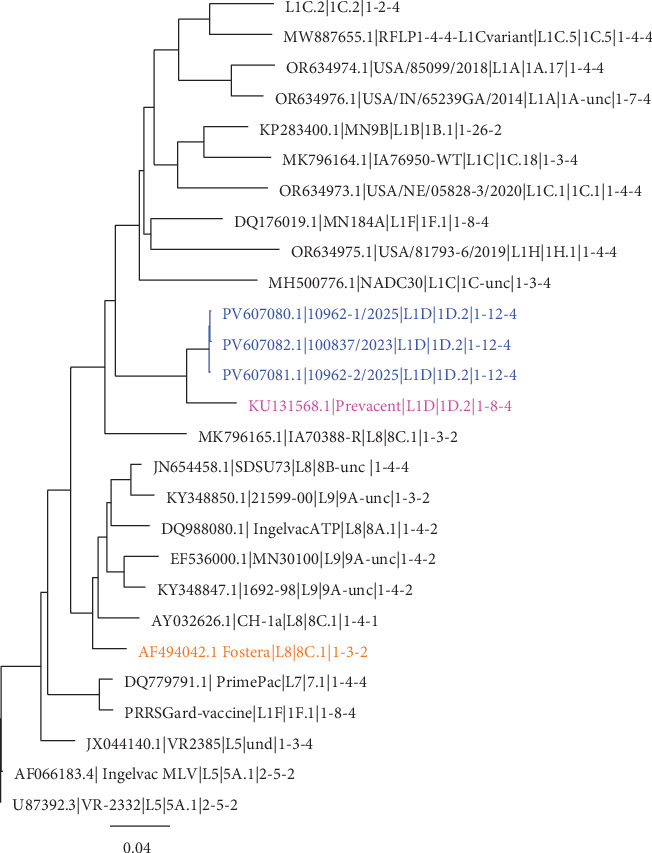
Whole PRRSV genome neighbor-joining phylogenetic tree representing the recombinant isolates PV607082, the parental strain Prevacent PRRS (purple), and Fostera PRRS (orange). The phylogenetic tree is rooted by the PRRSV-2 referent strain VR-2332 and includes additional PRRSV-2 wild-type strains and PRRSV-modified live vaccines commercially available in the United States, and other PRRSV-2 wild-type strains are presented as references. Sequences are labeled as GenBank Number|Isolate name|Lineage|Variant|RFLP. PRRSGard and L1C.2 sequences do not have GenBank numbers. The variant abbreviation “unc” stands for unclassified. Lineage, variant, and RFLP are determined based on the ORF5 gene.

Both plaque purifications were successfully recovered, and whole-PRRSV genomes recovered by NGS had 99.87% and 99.82% nucleotide identity with the whole genome recovered from sera, confirming the presence of the same virus (Figure 3). One plaque-purified PRRSV genome comprised 14,949 bp, position 37–14,985 of alignment with the sera-recovered whole genome, and had 20 nucleotide differences. The other PRRSV genome comprised 14,959 bp, positions seven to 14,965 of alignment with the sera-recovered whole genome, and had 27 nucleotide differences. Four nucleotide changes were the same from the sera and the two plaque-purified recovered genomes corresponding to a change from a C to T at positions 8263 and 14,711, A to G at position 9290, and a T to C at 12,613 of the alignment. No evidence of a Fostera-like virus contig was recovered from the plaque-purified materials, as occurred from the sera.

**Figure 4:**
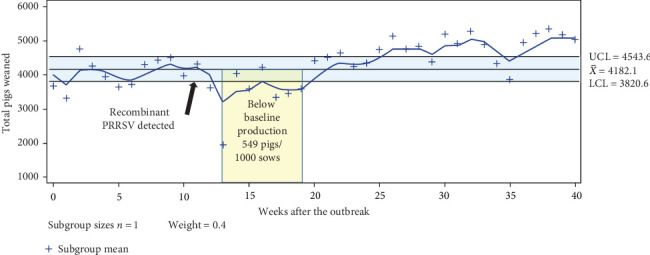
Exponential weighted moving average for total piglets weaned. The yellow shaded area represents the identified period with detected changes in lowering piglet productivity after the detection of recombinant virus.

A drop in total pigs weaned was first identified by the EWMA monitoring in the week of November 11^th^, 2023, right after the reporting of clinical signs in piglets and detection of a recombinant PRRSV. The production losses remained for 7 weeks and were calculated at 549 pigs per 1000 sows (Figure 4). There have been periodically PRRSV-positive samples coming from wean pig sera tested by RT-PCR up to April 16^th^, 2024, when the Sanger method recovered a sequence with 98.7% nucleotide identity with Fostera PRRS, 38 weeks after the first introduction of gilts in the sow farm, but piglets and sows were healthy. The farm had negative RT-PCR results until April 2025, when monitoring stopped.

**Figure 5:**
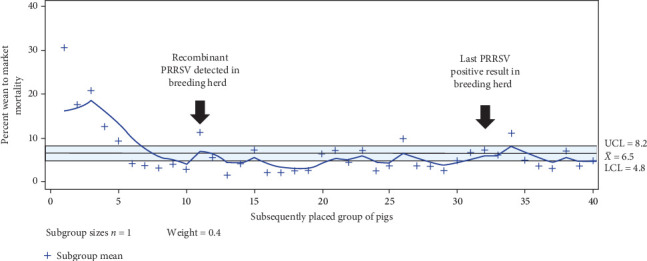
Exponential weighted moving average for wean-to-market mortality. The arrows indicate the time of recombinant virus detection and the last PRRSV-positive sample in the breeding herd.

The wean-to-market mortality monitored by EWMA indicated a transitory change from mortality from 3.85% to 11.18% for a placed group during the week of detection of a PRRSV recombination event in the breeding herd. Thereafter, the closeout mortality has been transiently changing from within monitoring levels to below EWMA monitoring lower confidence limit. There was a −0.46% mortality difference from before and after week 0 from a mean of 7.4 (May to July 2023) to 6.94% (August 2023 to April 2024) in the wean-to-market phase closeout mortality (*p* value 0.0103) (Figure 5).

The search for the ORF5 sequence in the SDRS database and up to the end of May 2025 revealed the detection of one sequence from April 4^th^, 2025, in the state of Iowa, which was reported to the SDRS project and had 99.8% nucleotide identity to PV607082. SDRS findings indicated a potential one-time point detection of the recombinant virus in the study breeding herd with no subsequent detection events in the same region where the farm is located. Pigs have been moved to the state of Iowa, and the additional detection has occurred 1 year after the herd eliminated the virus.

## 4. Discussion

The present work reported the emergence of an MLV-like derived recombinant strain. The identified production impact was losses of 549 piglets per 1000 sows during the event, with transitory but not significant losses in the wean-to-market phase. Notably, an increase in wean-to-market mortality observed in the first four placed groups following week 0 could have been associated with the ongoing Influenza A virus health challenge reported in the breeding herd.

Production measures like biocontainment practices intended to reduce the spread of PRRSV across piglets were reinforced on the farm. Interventions including no cross-fostering of piglets, changing of needles between sows and litters, needle-free prefarrow vaccination, focus on culling sows that appeared clinically affected and routine performance of general cleanliness were targeted measures. The GDU was emptied to create a break in the presumed circulation that could be occurring with the continued introduction of new animals. The implemented production practices contributed to reducing the circulation of this virus and avoiding a resurgence in the clinical presentation of the recombinant virus. The enforcement of biosecurity and biocontainment practices may also have contributed to improving the total pigs weaned per week reported after week 25 (Figure 4) and lower wean-to-market mortality (Figure 5).

The findings through the SDRS database indicate that the natural selection of recombinant virus failed to establish in the swine population, either due to animal immunity conferred through vaccine's ability to block this virus, the mild clinical presentation of this virus, or biosecurity and biocontainment practices routinely implemented across farms to contain the spread of pathogens. Nevertheless, wean-to-finish placed piglets were considered PRRSV positive, and no follow-up diagnostic test was conducted for PRRSV surveillance. The recombination breakpoint within the ORF5 generated a unique virus, and even though the recombinant virus could have been circulating in the wean-to-finish placed animals from the same system, no diagnostic testing was performed to further identify the virus. A similar ORF5 sequence was detected in another state in April 2025, 1 year after its elimination from the breeding herd in April 2024. The lack of similar sequences in the SDRS indicated no other detection in farms from other production flows from the state of Indiana or neighboring states. The detection of another similar virus in Iowa in 2025 could be due to the movement of positive animals to this region and the potential ability of the virus to keep circulating, or due to the occurrence of another recombination event between the two viruses. No further information beyond what was identified in the SDRS Blast tool was available for the 2025 sequence.

In the present report, the recombinant virus caused clinical problems in the breeding herd but did not cause any significant impacts on the downstream pig flow in terms of mortality and reduced pig performance. The MLV-derived virus had a mild production impact with 85% less piglet losses, that is, 549 pigs compared with 3675 piglets/1000 sows in outbreaks with wild-type strains [40]. Also, the herd took 7 weeks to return to baseline production, which is ~15 weeks shorter than outbreaks with wild-type strains [40]. PRRSV-1 MLV emergent recombinant virus has been demonstrated to produce clinical signs in breeding herds [22], but not necessarily as aggressive as some wild-type PRRSV-1 virus strains [41, 42]. A laboratory-created wild-type and vaccine recombinant virus has demonstrated under experimental trials a less clinically aggressive phenotype compared to the wild-type strain, but not as attenuated as the vaccine virus [43]. However, recent PRRSV recombinations between wild-type viruses have created very aggressive strains, an example being the PRRSV-2 L1C.5 that emerged in the United States in 2020 and the PRRSV-1 “Rosalia” in Spain, threatening swine production [25, 44, 45]. The information generated in this observational study indicates that recombinant PRRSV derived entirely from MLV strains is potentially less aggressive and can be removed from the breeding herd promptly compared to recombinant virus derived from MLV vaccine virus with wild-type strains or between wild-type viruses. Nevertheless, this observational study has limitations, which include only one experience with an MLV recombinant virus without replicates to demonstrate similar clinical outcomes, posing challenges for external validity.

In the present case, the usage of MLV followed label recommendations of being used in healthy animals, but the recombination arose due to the incorrect administration of a different MLV vaccine in the herd. The recombination event was plausible due to the circulation of two MLV vaccine viruses in the herd. There are production practices to consider, such as avoiding the rotation of different MLV vaccines in the herd and keeping vaccination protocols between GDU, breeding herd, and downstream flow consistent. These practices can be implemented to reduce such mistakes that could lead to the emergence of new viruses. If a need to vaccinate piglets with a different MLV is being used in the herd, it would be opportune to take place on the destination site to avoid the circulation of the second virus on top of production pyramid farms. Another practice that can be used to avoid recombination events is to avoid mixing viremic animals infected by two different PRRSV strains and avoid intentional exposure of an animal to two viruses at the same time, either by MLV vaccine usage or live virus inoculum (LVI). If using an LVI, test the inoculum by NGS to determine whether multiple PRRSV strains or other animal health-threatening pathogens are present; if the presence of more than one PRRSV or other pathogens is detected, avoid using the LVI material.

Recombination events have been associated with the genetic evolution of PRRSV [19, 21]. Recombination events can occur at different breakpoints, with multiple recombination events involving the MLV vaccines being reported in North America. A recombination between Fostera PRRS and a wild-type virus has been reported to occur on the nsp5 encoding region of the ORF1a gene [29], a different location from the one reported in this study. Another study reported a recombination event between Prevacent PRRS and Ingelvac PRRS MLV (GenBank AF066183), a PRRSV-2-derived MLV belonging to ORF5-based Lineage 5, Variant 5A.1 RFLP 2-5-2, occurring at the nsp8 encoding region [27], a similar region to one of the breakpoints reported in this study. A recombination between Prevacent PRRS and lineage 1 wild-type virus was reported as occurring within the ORF6 gene [28]. Another recombinant between an MLV and wild-type virus around or within ORF6 was also reported; however, the precise location and parental virus were not identified [26]. Additional wild-type and vaccine recombination events have also been reported in North America [23, 46]. Intra and interlineage PRRSV recombination has been reported to occur at variable frequency levels based on whole genomes [47, 48, 21] or using ORF5 sequences [49]. Increased access to diagnostic techniques and bioinformatics capabilities has enhanced our understanding of how reassortment events drive viral evolution over time and help explain acute changes in diagnostic presentation.

## Data Availability

The assembled PRRSV sequence has been deposited in GenBank under accession IDs PV607080, PV607081, and PV607082.

## References

[1] Zimmerman J. J., Dee S. A., Holtkamp D. J., Zimmerman J. J., Karriker L. A., Ramirez A., Schwartz K. J., Stevenson G. W., Zhang J. (2019). Porcine Reproductive and Respiratory Syndrome Viruses (Porcine Arteriviruses). *Diseases of Swine*.

[2] Carman S., Sanford S. E., Dea S. (1995). Assessment of Seropositivity to Porcine Reproductive and Respiratory Syndrome (PRRS) Virus in Swine Herds in Ontario—1978 to 1982. *Canadian Veterinary Journal-Revue Veterinaire Canadienne*.

[3] Benfield D. A., Nelson E., Collins J. E. (1992). Characterization of Swine Infertility and Respiratory Syndrome (SIRS) Virus (Isolate ATCC VR-2332). *Journal of Veterinary Diagnostic Investigation*.

[4] Collins J. E., Benfield D. A., Christianson W. T. (1992). Isolation of Swine Infertility and Respiratory Syndrome Virus (Isolate ATCC VR-2332) in North America and Experimental Reproduction of the Disease in Gnotobiotic Pigs. *Journal of Veterinary Diagnostic Investigation*.

[5] Dea S., Bilodeau R., Athanaseous R., Sauvageau R., Martineau G. (1992). PRRS Syndrome in Quebec: Isolation of a Virus Serologically Related to Lelystad Virus. *Veterinary Record*.

[6] Meulenberg J. J., Hulst M. M., de Meijer E. J. (1993). Lelystad Virus, the Causative Agent of Porcine Epidemic Abortion and Respiratory Syndrome (PEARS), Is Related to LDV and EAV. *Virology*.

[7] Murtaugh M. P., Elam M. R., Kakach L. T. (1995). Comparison of the Structural Protein Coding Sequences of the VR-2332 and Lelystad Virus Strains of the PRRS Virus. *Archives of Virology*.

[8] Terpstra C., Wensvoort G., Pol J. M. A. (1991). Experimental Reproduction of Porcine Epidemic Abortion and Respiratory Syndrome (mystery Swine Disease) by Infection With Lelystad Virus: Koch’s Postulates Fulfilled. *Veterinary Quarterly*.

[9] Trevisan G., Linhares L. C. M., Crim B. (2019). Macroepidemiological Aspects of Porcine Reproductive and Respiratory Syndrome Virus Detection by Major United States Veterinary Diagnostic Laboratories Over Time, Age Group, and Specimen. *PLoS One*.

[10] Wensvoort G., Terpstra C., Pol J. M. (1991). Mystery Swine Disease in the Netherlands: The Isolation of Lelystad Virus. *Veterinary Quarterly*.

[11] Holtkamp D. J., Kliebenstein J. B., Neumann E. J. (2013). Assessment of the Economic Impact of Porcine Reproductive and Respiratory Syndrome Virus on United States Pork Producers. *Journal of Swine Health and Production*.

[12] Osemeke O. H., Silva G. S., Corzo C. (2024). Updating the Productivity and Economic Cost of PRRSV in the US.

[13] Mussell A., Oginskyy A., Grier K. (2011). *A Risk, Benefit, Strength, Weakness, Opportunity and Threat Analysis for the Control and Possible Eradication of Porcine Reproductive and Respiratory Syndrome (PRRS) Virus Within the Canadian Swine Herd*.

[14] Nathues H., Alarcon P., Rushton J. (2017). Cost of Porcine Reproductive and Respiratory Syndrome Virus at Individual Farm Level–An Economic Disease Model. *Preventive Veterinary Medicine*.

[15] Nieuwenhuis N., Duinhof T. F., van Nes A. (2012). Economic Analysis of Outbreaks of Porcine Reproductive and Respiratory Syndrome Virus in Nine Sow Herds. *Veterinary Record*.

[16] Dolan P. T., Whitfield Z. J., Andino R. (2018). Mechanisms and Concepts in RNA Virus Population Dynamics and Evolution. *Annual Review of Virology*.

[17] Murtaugh M. P., Yuan S., Nelson E. A., Faaberg K. S. (2002). Genetic Interaction Between Porcine Reproductive and Respiratory Syndrome Virus (PRRSV) Strains in Cell Culture and in Animals. *Journal of Swine Health and Production*.

[18] Mötz M., Stadler J., Kreutzmann H. (2023). A Conserved Stem-Loop Structure Within ORF5 is a Frequent Recombination Hotspot for Porcine Reproductive and Respiratory Syndrome Virus 1 (PRRSV-1) with a Particular Modified Live Virus (MLV) Strain. *Viruses*.

[19] Yuan S., Nelsen C. J., Murtaugh M. P., Schmitt B. J., Faaberg K. S. (1999). Recombination Between North American Strains of Porcine Reproductive and Respiratory Syndrome Virus. *Virus Research*.

[20] Eclercy J., Renson P., Lebret A. (2019). A Field Recombinant Strain Derived From Two Type 1 Porcine Reproductive and Respiratory Syndrome Virus (PRRSV-1) Modified Live Vaccines Shows Increased Viremia and Transmission in SPF Pigs. *Viruses*.

[21] Guo J., Liu Z., Tong X. (2021). Evolutionary Dynamics of Type 2 Porcine Reproductive and Respiratory Syndrome Virus by Whole-Genome Analysis. *Viruses*.

[22] Kvisgaard L. K., Kristensen C. S., Ryt-Hansen P. (2020). A Recombination Between Two Type 1 Porcine Reproductive and Respiratory Syndrome Virus (PRRSV-1) Vaccine Strains Has Caused Severe Outbreaks in Danish Pigs. *Transboundary and Emerging Diseases*.

[23] Lalonde C., Provost C., Gagnon C. A. (2020). Whole-Genome Sequencing of Porcine Reproductive and Respiratory Syndrome Virus From Field Clinical Samples Improves the Genomic Surveillance of the Virus. *Journal of Clinical Microbiology*.

[24] Liu D., Zhou R., Zhang J. (2011). Recombination Analyses Between Two Strains of Porcine Reproductive and Respiratory Syndrome Virus in Vivo. *Virus Research*.

[25] Pamornchainavakul N., Kikuti M., Paploski I. A. D. (2022). Measuring How Recombination Re-Shapes the Evolutionary History of PRRSV-2: A Genome-Based Phylodynamic Analysis of the Emergence of a Novel PRRSV-2 Variant. *Frontiers in Veterinary Science*.

[26] Risser J., Ackerman M., Evelsizer R., Wu S., Kwon B., Hammer J. M. (2021). Porcine Reproductive and Respiratory Syndrome Virus Genetic Variability a Management and Diagnostic Dilemma. *Virology Journal*.

[27] Trevisan G., Magstadt D., Woods A. (2023). A Recombinant Porcine Reproductive and Respiratory Syndrome Virus Type 2 Field Strain Derived From Two PRRSV-2-Modified Live Virus Vaccines. *Frontiers in Veterinary Science*.

[28] Trevisan G., Zeller M., Li G., Zhang J., Gauger P., Linhares D. C. L. (2022). Implementing a User-Friendly Format to Analyze PRRSV Next-Generation Sequencing Results and Associating Breeding Herd Production Performance With Number of PRRSV Strains and Recombination Events. *Transboundary and Emerging Diseases*.

[29] Wang A., Chen Q., Wang L. (2019). Recombination Between Vaccine and Field Strains of Porcine Reproductive and Respiratory Syndrome Virus. *Emerging Infectious Diseases*.

[30] Yim-Im W., Anderson T. K., Paploski I. A. D. (2023). Refining PRRSV-2 Genetic Classification Based on Global ORF5 Sequences and Investigation of Their Geographic Distributions and Temporal Changes. *Microbiology Spectrum*.

[31] VanderWaal K., Pamornchainavakul N., Kikuti M. (2025). PRRSV-2 Variant Classification: A Dynamic Nomenclature for Enhanced Monitoring and Surveillance. *MSphere*.

[32] Wesley R. D., Mengeling W. L., Lager K. M., Clouser D. F., Landgraf J. G., Frey M. L. (1998). Differentiation of a Porcine Reproductive and Respiratory Syndrome Virus Vaccine Strain from North American Field Strains by Restriction Fragment Length Polymorphism Analysis of ORF 5. *Journal of Veterinary Diagnostic Investigation*.

[33] Gauger P., Harmon K. PRRS CLAMP: Molecular Diagnostic Tools to Distinguish Wild-Type and Vaccine Strains of PRRSV.

[34] Zhang J., Zheng Y., Xia X. Q. (2017). High-Throughput Whole Genome Sequencing of Porcine Reproductive and Respiratory Syndrome Virus From Cell Culture Materials and Clinical Specimens Using Next-Generation Sequencing Technology. *Journal of Veterinary Diagnostic Investigation*.

[35] Katoh K., Standley D. M. (2013). MAFFT Multiple Sequence Alignment Software Version 7: Improvements in Performance and Usability. *Molecular Biology and Evolution*.

[36] Martin D. P., Murrell B., Golden M., Khoosal A., Muhire B. (2015). RDP4: Detection and Analysis of Recombination Patterns in Virus Genomes. *Virus Evolution*.

[37] Lole K. S., Bollinger R. C., Paranjape R. S. (1999). Full-Length Human Immunodeficiency Virus Type 1 Genomes From Subtype C-Infected Seroconverters in India, With Evidence of Intersubtype Recombination. *Journal of Virology*.

[38] Montgomery D. (2009). *Introduction to Statistical Quality Control*.

[39] Chandra S., Cezar G., Rupasinghe K. (2025). Harnessing Sequencing Data for Porcine Reproductive and Respiratory Syndrome Virus (PRRSV): Tracking Genetic Evolution Dynamics and Emerging Sequences in US Swine Industry. *Frontiers in veterinary science*.

[40] Paiva R., Rademacher C., Peterson T. (2024). Description of Practices Adopted in Response to Porcine Reproductive and Respiratory Syndrome Outbreaks Among Breeding Herds in the United States From 2019-2021. *Journal of Swine Health and Production*.

[41] Genís S., Kvisgaard L. K., Larsen L. E., Taylor L. P., Calvert J. G., Balasch M. (2020). Assessment of the Impact of the Recombinant Porcine Reproductive and Respiratory Syndrome Virus Horsens Strain on the Reproductive Performance in Pregnant Sows. *Pathogens*.

[42] Kvisgaard L. K., Larsen L. E., Kristensen C. S., Paboeuf F., Renson P., Bourry O. (2021). Challenge of Naïve and Vaccinated Pigs with a Vaccine-Derived Recombinant Porcine Reproductive and Respiratory Syndrome Virus 1 Strain (Horsens Strain). *Vaccines (Basel)*.

[43] Wang Y., Liang Y., Han J. (2008). Attenuation of Porcine Reproductive and Respiratory Syndrome Virus Strain MN184 Using Chimeric Construction With Vaccine Sequence. *Virology*.

[44] Martín-Valls G. E., Cortey M., Allepuz A., Illas F., Tello M., Mateu E. (2022). Description of a New Clade Within Subtype 1 of Betaarterivirus suid 1 Causing Severe Outbreaks in Spain. *Microbiology Resource Announcements*.

[45] Trevisan G., Li G., Moura C. A. A. (2021). Complete Coding Genome Sequence of a Novel Porcine Reproductive and Respiratory Syndrome Virus 2 Restriction Fragment Length Polymorphism 1-4-4 Lineage 1C Variant Identified in Iowa, USA. *Microbiology Resource Announcements*.

[46] Rawal G., Krueger K. M., Yim-Im W. (2023). Development, Evaluation, and Clinical Application of PRRSV-2 Vaccine-Like Real-Time RT-PCR Assays. *Viruses*.

[47] Cui X., Xia D., Huang X. (2022). Analysis of Recombinant Characteristics Based on 949 PRRSV-2 Genomic Sequences Obtained From 1991 to 2021 Shows That Viral Multiplication Ability Contributes to Dominant Recombination. *Microbiology Spectrum*.

[48] Cui X.-Y., Xia D.-S., Huang X.-Y. (2022). Recombinant Characteristics, Pathogenicity, and Viral Shedding of a Novel PRRSV Variant Derived From Twice Inter-Lineage Recombination. *Veterinary Microbiology*.

[49] Shi M., Lemey P., Singh Brar M. (2013). the Spread of Type 2 Porcine Reproductive and Respiratory Syndrome Virus (PRRSV) in North America: A Phylogeographic Approach. *Virology*.

